# Maximizing the benefits of antiretroviral therapy for key affected populations

**DOI:** 10.7448/IAS.17.1.19320

**Published:** 2014-07-18

**Authors:** Ian R Grubb, Sarah W Beckham, Michel Kazatchkine, Ruth M Thomas, Eliot R Albers, Mauro Cabral, Joep Lange, Stefano Vella, Manoj Kurian, Chris Beyrer, for the IAS Treatment for Key Affected Populations Working Group

**Affiliations:** 1 Center for Public Health and Human Rights, Johns Hopkins University Baltimore, MD, USA; 2 Department of Epidemiology, Johns Hopkins University, Baltimore, MD, USA; 3 UN, Geneva, Switzerland; 4 Global Network of Sex Work Projects, Edinburgh, United Kingdom; 5 International Network of People Who Use Drugs, London, United Kingdom; 6 Global Action for Trans* Equality, Buenos Aires, Argentina; 7 Amsterdam Institute of Global Health and Development, Amsterdam, The Netherlands; 8 Department of Therapeutics, HIV, Hepatitis and Global Health, Istituto Superiore di Sanità, Rome, Italy; 9 International AIDS Society, Geneva, Switzerland

**Keywords:** treatment, HIV/AIDS, human rights

## Abstract

**Introduction:**

Scientific research has demonstrated the clinical benefits of earlier initiation of antiretroviral treatment (ART), and that ART can markedly reduce HIV transmission to sexual partners. Ensuring universal access to ART for those who need it has long been a core principle of the HIV response, and extending the benefits of ART to key populations is critical to increasing the impact of ART and the overall effectiveness of the HIV response. However, this can only be achieved through coordinated efforts to address political, social, legal and economic barriers that key populations face in accessing HIV services.

**Discussion:**

Recent analyses show that HIV prevalence levels among key populations are far higher than among the general population, and they experience a range of biological and behavioural factors, and social, legal and economic barriers that increase their vulnerability to HIV and have resulted in alarmingly low ART coverage. World Health Organization 2014 consolidated guidance on HIV among key populations offers the potential for increased access to ART by key populations, following the same principles as for the general adult population. However, it should not be assumed that key populations will achieve greater access to ART unless stigma, discrimination and punitive laws, policies and practices that limit access to ART and other HIV interventions in many countries are addressed.

**Conclusions:**

Rights-based approaches and investments in critical enablers, such as supportive legal and policy environments, are essential to enable wider access to ART and other HIV interventions for key populations. The primary objective of ART should always be to treat the person living with HIV; prevention is an important, additional benefit. ART should be provided only with informed consent. The preventive benefits of treatment must not be used as a pretext for failure to provide other necessary HIV programming for key populations, including comprehensive harm reduction and other prevention interventions tailored to meet the needs of key populations. An end to AIDS is only possible if we overcome the barriers of criminalization, stigma and discrimination that remain key drivers of the HIV epidemics among key populations.

## Introduction

During the past 20 years, antiretroviral treatment (ART) has achieved remarkable success in delaying HIV-related disease progression and reducing AIDS-related mortality. In addition to the individual clinical benefits [1], early initiation of ART also has been shown to reduce transmission [2] and decrease HIV acquisition risk at the population level [3]. These results are mobilizing the global HIV/AIDS community to seek accelerated scale-up of earlier ART as both a treatment and a prevention strategy. The effort is accompanied by new, comprehensive guidelines on treatment from the World Health Organization (WHO) [4] indicating earlier initiation of treatment. The new guidelines markedly increase the number of people potentially eligible for this life-saving therapy. While resources and operational issues remain an enormous challenge to maximizing the benefits of ART, the fundamental concept that universal access to ART is a key next step in the global response to HIV/AIDS is now widely accepted. Universal access is an essential goal both on human rights and on scientific grounds.

Achieving universal access in the coming years necessarily involves addressing the needs of key affected populations (KP). KP are defined by the International AIDS Society (IAS) as having disproportionate burdens of HIV infection, *as well as* low levels of access to essential HIV services. This includes gay, bisexual and other men who have sex with men (MSM), sex workers of all genders, people who inject drugs (PWID) and transgender people. In some settings, prisoners and migrants may also be particularly vulnerable to HIV and to exclusion from HIV services. While extending the benefits of ART to these highly marginalized populations will ultimately increase the impact of ART and the effectiveness of the HIV response, it also involves addressing with urgency a wide range of social, political, legal and economic barriers that continue to limit their access to healthcare, including HIV prevention and treatment. A further challenge to HIV responses for KP is the lack of disaggregated data, and this is particularly true for adolescents who may be within these groups.

Despite gaps in current knowledge, it is clear that social and cultural attitudes, including stigma, discrimination and punitive laws, policies and practices, present barriers to access. To benefit from ART, people living with HIV/AIDS (PLHA) must first know their status, and this requires voluntary HIV testing and linkage to care in contexts of safety, dignity, and confidentiality. However, in too many countries and contexts, KP are discriminated against, stigmatized in communities and in healthcare settings, criminalized, and excluded – or exclude themselves – from essential HIV services.

In 2014, KP are arguably facing *rising* threats to accessing care. The recent wave of anti-homosexual court decisions and legislation in Russia, India, Nigeria and Uganda underscores the threats to universal access faced by these populations and is illustrative of the fundamental challenges involved in extending the benefits of ART access to those who need it. It cannot be assumed that because the scientific community has now reached consensus on the benefits of earlier treatment initiation that this will become genuinely available to KP without extraordinary effort. This article, a joint undertaking of the KP Working Group and the Treatment as Prevention Working Groups of the IAS, aims to describe and analyse the challenges and barriers that KP face in accessing and using ART for both treatment and prevention, and it provides context to the new “Consolidated Guidelines” on HIV among key populations, which were released by WHO in July 2014.

## Disproportionate Burdens

KP experience disproportionately high disease burden and multiple risk factors in nearly all countries. It has long been assumed that KP represent a modest share of the epidemic globally, and that the HIV prevalence among them is largely confined to countries with low-level and concentrated epidemics. It is now increasingly recognized that KP and their sex partners not only represent most of the PLHA outside sub-Saharan Africa, but also a significant proportion of new infections in sub-Saharan Africa [5–8]. As HIV begins to spread to other populations, the distinction between concentrated and generalized epidemic settings is becoming less relevant. In many countries, however, coverage of HIV prevention interventions for KP is grossly inadequate, and access to health services, including HIV testing and ART, is limited by discrimination, stigma, punitive laws and policies and lack of community empowerment.

### HIV burden and risk factors

The burden of HIV among KP in generalized epidemics has been poorly understood due to their limited representation in national surveillance data and the fact that they are criminalized and stigmatized in many settings. Table 1 highlights the HIV burden for MSM, sex workers, PWID and transgender people, where data are available, and lists some known risk factors and current intervention recommendations. This is not a comprehensive review of the evidence; rather, it gives a sense of the epidemic's magnitude among KP. Common to all the populations, and not reflected in the table, are the structural risk factors they face, such as criminalization, discriminatory and punitive laws and policies, and pervasive stigmatization, all of which limit their abilities to protect themselves from HIV acquisition and transmission. These structural risk factors and barriers are too numerous to list here; specific examples include condoms being used as evidence for prostitution, the lack of condoms in prisons due to sodomy laws, and the lack of methadone due to laws against its use in the Russian Federation [9–12].

**Table 1:** HIV burden and risk factors among key affected populations

Population	Prevalence	Incidence	OR	Major risk factors	Existing interventions
MSM	15% (N. America, S. and SE Asia) 18% (sub-Saharan Africa) 25% (Caribbean) [14]	6.8/100 py (Kenya and South Africa) [13] 7.7% (Thailand) [15] 10% (China) [16]	19.3^a^ [14]	Unprotected receptive anal intercourse; high number of male partner frequency; high number of lifetime male partners; injection drug use; high viral load in index partner; non-injection drug use (stimulants); network-level effects [5]	Behavioural: reduce alcohol and drug use; reduce number of partners; increase condom use and adherence to ART Biomedical: ART; oral PreP Structural: decriminalization of homosexuality and “sodomy”; accessible and acceptable health services [9]
SW	11.8% (50 countries) 36.9% (sub-Saharan Africa) [18]	3.6/100 py (Cambodia) [17] 13.9/100 py (Tanzania) [19]	13.5^b^ [18]	High-risk sexual exposures; high number of partners; high prevalence of STI; poverty; gender inequity; sexual violence [10]	Behavioural: condom use; HIV testing Biomedical: STI diagnosis and treatment; ART; HBV immunization Structural: decriminalization of sex work; anti-discrimination laws; accessible and acceptable health services; addressing violence; empowerment and community mobilization [10]
PWID	18.8% worldwide 9–22% in 6 highest burden countries [22]	4.5/100 py (Russia) [20] 8.01/100 py (India) [23]	No data	Reusing injecting equipment; detention and incarceration [21]	Behavioural: HIV testing; condom promotion for PWID and their partners; tailored education and communication Biomedical: opioid substitution therapy; ART; STI treatment; prevention and treatment of TB; prevention, vaccination, and treatment of viral hepatitis Structural: needle and syringe programmes [11]
Transgender women	27.7% (US) [24] 27.3% (TG sex workers, 13 countries) 14.7% (13 countries) [25] 19.1% (15 countries) [12]	No data	4^c^ [25]	Unprotected receptive anal sex; network-level effects (sexual networks overlap with MSM populations) [12]	Behavioural: increase condom and lubricant use; HIV testing Biomedical: PrEP; early ART; microbicides Structural: decriminalization of “cross-dressing” and “sodomy”; anti-discrimination laws; legal recognition of gender identity; gender-affirming health services; community engagement and empowerment; peer outreach [26]

a OR compared to general male population, low- and middle-income countries

b OR compared to general female population, low- and middle-income countries

c transgender sex workers versus female sex workers.

Globally, epidemics of HIV are expanding among MSM with consistently high incidence rates [26]. Data from multiple continents show consistently high incidence rates, particularly among the youngest age groups, and in many high-income settings, overall epidemic trends are in decline *except* among MSM [6]. MSM now account for more than one-third of new infections in China [27], and may constitute half or more of all new infections in Asia by 2020 [28]. HIV prevention efforts among MSM have yielded many successes but have been insufficient, and in sub-Saharan Africa, they are extremely rare [29]. Biomedical and behavioural combination interventions are increasingly emphasized [30].

Sex workers have been reported to be at high risk of HIV infection in many settings. Especially high prevalence among FSW has been reported in sub-Saharan Africa, from 24% (Rwanda) to 71% (Malawi), and FSW are estimated to account for large proportions of new infections [18]. Structural and behavioural interventions have successfully decreased HIV transmission among FSW, and in most settings, consistent condom use and HIV testing are higher among FSW than other women [31]. Still, on-going HIV prevention programmes cover fewer than half of sex workers worldwide [32]. Relatively little research has been undertaken among male and transgender sex workers, but they are likely at increased risk [33].

There are an estimated 16 million PWID worldwide, of whom three million are living with HIV. They account for roughly one-third of all HIV infections outside sub-Saharan Africa, and HIV epidemics among PWID are increasing across Eastern Europe and Central Asia [22]. PWID are also at high risk for incarceration and tuberculosis (TB). Additionally, PWID need services for drug dependence treatment, which can have easily manageable interactions with HIV, TB or hepatitis C virus (HCV) medications [34].

Transgender women are people who are assigned male gender at birth but identify as women. They have long been known to be at high risk of HIV infection, but national surveillance systems have not captured HIV data among them, and many transgender women are unaware of their status. The severity of transgender people's disease burden is consistent across all regions, notwithstanding the widely varying cultural, social, political and legal contexts in which they live. Data on HIV burden among transgender men are limited, but recent studies challenge the assumption that their risks are low [35,36].

HIV prevalence among prisoners ranges from 0% (Middle East) to 41.4% (South Africa) [37,38], and levels are usually several times higher than in outside communities [37]. This is partly due to high rates of incarceration among PWID [39]. An estimated one in seven PLHA enters a correctional facility each year in the United States [40], and injection drug use in African prisons has reportedly been increasing [41]. In Asia, PWID and other KP are arbitrarily detained for lengthy periods, and are subject to forced labour and other abuses, including lack of access to healthcare [42]. Risk factors include reusing injection equipment, untreated mental illness, lower socioeconomic status, belonging to an ethnic minority [37,38], tattooing and piercing, violence and rape [43]. High rates of other sexually transmitted infections (STI), HCV and TB are also widely reported [32,43–46]. Available interventions and technologies are sufficient to control the HIV epidemic among prisoners, but harmful policies, laws and policing practices, as well as low access to services, are the principle barriers to achieving effective impact.

Given the diverse contexts in which migrants live, it is difficult to summarize their HIV burden, even where data exist. Migrants from high-prevalence countries may have higher likelihoods of HIV infection than host country nationals in some settings, while elsewhere they may be moving from lower to higher prevalence contexts. HIV risk can be exacerbated by low access to services, legal status, language barriers and illiteracy, unemployment, laws and practices that expel PLHA, mobility, and post-conflict mental health issues, and internal migrants can also face many of these same issues [47–50]. Frequently, migrants are blamed for and unjustly stigmatized as spreading HIV [47], and they may also be more frequently prosecuted for transmission and exposure than other populations [51]. Migrants need standard protection under the law regardless of citizenship, voluntary rather than compulsory HIV testing and an end to punitive laws against migrants and PLHA [51].

### ART coverage for KP

While the benefits of ART are established, and treatment adherence for KP is comparable to that of other adults in similar treatment contexts, given appropriate structural support [34,52,53], there are major gaps in the knowledge about ART coverage among KP. This is partly because national data systems rarely disaggregate data and because of concerns that classifying people may lead to human rights violations. Nevertheless, the available data reveal significant inequities in coverage for all KP.

For MSM, ART coverage reportedly ranges from 14% (low-income countries) to 51% (high-income countries) [54]. The percentage of MSM who know their HIV status is low, indicating that ART coverage is also low [55]. Studies on ART and transmission dynamics among MSM suggest a complex and challenging picture [26,56], but preliminary data suggest that treatment may be as effective for MSM couples as for heterosexual ones [57]. Efforts to increase coverage should also address such issues and other barriers to maximize benefit. A modelling exercise estimated that significantly increased investments and coverage of ART for MSM could avert 75,000 infections in one year alone [58].

Among sex workers, knowledge of HIV status can be low, ranging from 16% (Asia) to 40% (sub-Saharan Africa), which suggests that ART coverage is also low [49]. The data that do exist indicate that FSW are less likely than other women to receive timely and adequate HIV treatment and care [59–61]. Significant barriers remain, even in contexts of extensive efforts to scale up ART among them [62]. Modelling suggests some prevention benefit of earlier treatment initiation for FSW [63], and for those in serodiscordant relationships with primary partners, treatment as prevention has high biological and clinical plausibility. Despite low levels of ART access, FSW accessing ART in Kenya were reported to have good treatment outcomes and no evidence of increased sexual risk behaviours [64]. One study has estimated that expanding access to ART for FSW in Kenya could reduce the number of FSW acquiring HIV by 25% [65].

For PWID, early treatment initiation, in combination with an evidence-based package of other preventive interventions, can be highly effective in reducing mortality and morbidity and controlling HIV transmission [21,66]. Despite this, an estimated 4% of PLHA who inject drugs worldwide were receiving ART, compared to 18% among all PLHA [67]. In the five countries with the largest HIV epidemics among PWID, PWID were 67% of HIV cases, but only 25% of those receiving ART [39]. Slightly increased levels of ART access for PWID were reported in three high-burden countries in 2010–2012 [22]. Many countries know that they have a high prevalence of HIV-positive PWID, but they do not attempt to measure ART coverage; estimates were available for only 10 out of 21 countries studied and ranged from 0.06% (Afghanistan) to 22% (Bangladesh) [68]. Furthermore, PWID may be less likely to be given ART in healthcare settings. More than a quarter of healthcare providers in Canada and the United States reported that they would defer ART for eligible people if they injected drugs [69], and a prospective cohort study in Ukraine found that pregnant PWID were less likely to receive ART than their counterparts who did not inject drugs [70].

As gender disaggregation does not take account of transgender status, there are virtually no ART coverage data for transgender people. A US study found that transgender women were less likely to have received ART compared to non-trans participants (59% vs. 82%) and that transgender women have higher HIV-related mortality and higher community viral load compared to non-trans people [71]. Another study of transgender women on ART in the United States found that the women were less likely to report good adherence, and they reported less confidence in their abilities to integrate treatment regimens into their daily lives and significantly fewer positive interactions with healthcare providers than other people [72]. A study in India found that widespread HIV stigma and prejudice based on gender identity and expression, sexual orientation and sex work motivated many transgender women to keep their HIV status secret, and they were powerful disincentives to access ART [73].

Data on ART coverage among prisoners are rare, though coverage is likely very low, as evidenced by poor coverage for other high-prevalence infections such as HCV and TB, as well as widespread human rights abuses in prisons worldwide. Prison health is frequently disregarded because Ministries of Interior or Justice, rather than Ministries of Health, are responsible for prison health services. In Uganda, for example, HIV prevalence in prisoners is double that in the general population, but ART is provided in only one out of 223 prisons [74]. Few countries implement comprehensive HIV and HCV prevention, treatment and care programmes in prisons, often because of ideological objections or denial about sex and drug use among prisoners, and many fail to adequately link health services in prisons to national programmes [75]. Abuses are starkly evident in the notorious detention centres in several Asia countries in which KP and others are arbitrarily detained for lengthy periods for “rehabilitation,” and are subject to forced labour and other abuses, including lack of access to adequate healthcare [42]. The high prevalence of infections in prison settings cannot be separated from broader public health concerns, and the fulfilment of the right to health of people in prisons needs to be seen as a part of state obligations to fulfil the right to health of the population as a whole.

Rates of ART coverage among migrants and refugees are unknown. The Global Commission on HIV and the Law estimated some 214 million international migrants worldwide, and 740 million internal migrants, in 2012 [76]. The Commission was clear in its findings for migrant populations: “In matters relating to HIV and the law, countries should offer the same standard of protection to migrants, visitors and residents who are not citizens as they do to their own citizens.” The reality is that migrants in most countries do not have equal access to healthcare or adequate standards of legal protection. Despite internationally recognized legal status [77], refugees often do not receive access to medical care on par with that received by citizens. Governments may cite migrants’ mobility or instability and the prioritization of care of its own citizens as reasons to deny ART to migrants [78]. People who have left their countries of origin do not lose the human right to healthcare, and migrants cannot be excluded from accessing services if the global HIV pandemic is to be controlled.

### Global guidance on antiretroviral therapies for treatment and prevention among KP

The WHO 2013 “Consolidated Guidelines on the Use of Antiretroviral Drugs for Treating and Preventing HIV Infection” [4] and new 2014 consolidated guidance on HIV among KP offer the potential for KP's increased access to HIV testing and counselling (HTC) and ART for both treatment and prevention.

#### ART

New clinical recommendations in the consolidated guidelines raised the threshold for treatment initiation to <500 CD4 cells/mm^3^, giving priority to individuals with severe or advanced HIV disease and those at <350 CD4 cells/mm^3^ [4]. Consistent with earlier WHO guidance [9,10], the 2013 guidelines recommend that ART initiation in KP should follow the same principles as for the general population (i.e. in general, initiated at a CD4 count of <500 cells/mm^3^). However, recommendations that ART should be initiated *regardless of CD4 count* for people in particular circumstances may apply to certain KP, including people who have HIV and active TB and people with an HIV-hepatitis B co-infection and severe chronic liver disease (this would include PWID, prisoners and migrants, among others), pregnant women (pregnant FSW living with HIV, women with HIV who inject drugs, prisoners and migrants, among others) and people in serodiscordant relationships (male same-sex couples, sex workers and their primary partners, and PWID and their primary partners, among others).

Additionally, WHO guidelines recommend fixed dose combinations including tenofovir as first-line regimens. While it has been extensively demonstrated that age, race, sex, educational level, socioeconomic status and a past history of alcoholism or drug use do not reliably predict suboptimal adherence, the indication of simpler combinations may reduce the risk of non-adherence, which can lead to resistance. Despite the WHO recommendations, the best nontoxic and easy-to-use ART may not be routinely available in resource-limited settings. Furthermore, specific populations like KP, due to structural factors, generally have difficult or very limited access to quality care and treatment options. Converging efforts, from the public and the private sector, must be made to ensure that the best drugs and the new drug classes, including the new HIV integrase inhibitors, become readily available worldwide and specifically for KP, to reach the levels of full and sustained HIV suppression which not only have a clinical and HIV transmission benefit, but also minimize the risk of a progressive spread of drug resistance.

WHO estimates that an additional 11 million PLHA globally will become eligible for ART under the 2013 guidelines. While it is unknown how many KP are newly eligible, it is plausible that the new guidelines could help to expand access to ART for both treatment and prevention for them. However, in many countries, the recommendations are unlikely to result in expanded access for KP unless special attention is paid to issues of equity, human rights and barriers that they face in accessing HIV and other health services.

#### HIV testing and counselling

The guidelines affirm earlier WHO guidance that in generalized epidemics, HTC should be routinely recommended for KP in all health facilities, and that in low and concentrated epidemics it should be considered in health facilities offering services for STI, hepatitis, TB and antenatal care, as well as in health services specifically for KP. Recognizing that many people from KP may not attend health facilities, the guidelines also recommend that community-based HTC should be available to KP in all epidemic settings, with appropriate confidentiality and informed consent.

#### Pre-exposure prophylaxis

The 2013 ART guidelines affirmed existing WHO guidance [55,79,80] that daily oral pre-exposure prophylaxis (PreP) may be considered as a possible intervention for uninfected partners in serodiscordant couples on a limited basis through “demonstration projects.” New WHO KP guidance in 2014 recommends that PreP be available as an option for serodiscordant couples and for men or transgender women who have sex with men, but there is no specific recommendation for other KP.

#### Post-exposure prophylaxis

Post-exposure prophylaxis (PEP) is the use of ART to prevent HIV infection after a single high-risk HIV exposure, and it has been used in some countries for MSM and sex workers. Since PEP must be started as soon as possible and must be taken for 28 days, it has had limited use as a prevention tool, but there is considerable evidence for its effectiveness. While PEP is an important prevention tool, it cannot substitute for other proven HIV prevention methods, such as consistent condom use or use of sterile injecting equipment [81], and PEP services may be an effective entry point for accessing these methods and other prevention services.

### Protecting rights and addressing barriers

Stigma, discrimination and punitive laws, policies and practices significantly limit access to ART and other HIV interventions [58,82,83]. Eighty countries outlaw same-sex activity [84]; more than 100 countries criminalize some aspect of sex work; many countries require registration of drug users or employ predominantly criminal justice – rather than public health – approaches to drug use; and effective drug dependence treatment is not legally available in many countries [85]. Criminalization of HIV transmission and failure to legally recognize transgender status also present barriers to ART access in many countries [71].

Data on KP remain very limited, including population size estimates and data relating to access and barriers to services. Data collection systems in countries need to more adequately address issues for KP, while always protecting individuals’ confidentiality and security. The international community must also more effectively address trade and intellectual property barriers to ART access, including TRIPS and “TRIPS-plus” provisions [51].

Rights-based approaches and investments in evidence-based critical enablers, such as supportive legal and policy environments, are essential to increase access to ART and other HIV interventions for KP. Evidence-based approaches can include strategic litigation [85]. Particular attention is needed to ensure that available services for KP are equipped to meet the needs of adolescents and young adults. Approaches must include measures to combat stigma and discrimination, mobilize and empower communities and increase the availability of non-judgmental, community-based services.

## Conclusions

Universal access to ART for those who need and want it has long been a core human rights principle of the HIV response, and it now has an expanded clinical and public health rationale. This article provides clear evidence that KP account for a significant proportion of the global burden of HIV, and that epidemics among KP are characterized by extraordinarily high prevalence levels and remarkably low levels of access to HIV treatment and prevention worldwide. Extending the benefits of ART to KP is critical to increasing the impact of ART and the overall effectiveness of the HIV response, but this can only be achieved through an extraordinary effort to address the barriers that KP face, including stigmatization, discrimination and punitive laws, policies and practices.

Communities at risk are justifiably sceptical that ART will be made available with the necessary equity, respect for human rights and non-discrimination that are required to ensure success. ART should always be offered in the context of comprehensive HIV care and prevention services, and only with informed consent. The primary objective of ART should always be to treat the person living with HIV; prevention is an important, secondary benefit. Additionally, governments must not use the preventive benefits of treatment as a pretext for failing to provide critical and comprehensive prevention interventions for KP, including condom promotion, behaviour change, comprehensive harm reduction for PWID, and community empowerment and other rights-based approaches. An end to AIDS is possible only if we overcome the barriers of criminalization, stigma and discrimination that remain key drivers of the epidemic.
